# The Role of Cytokines in Nephrotic Syndrome

**DOI:** 10.1155/2022/6499668

**Published:** 2022-02-09

**Authors:** Elham Ahmadian, Yalda Rahbar Saadat, Elaheh Dalir Abdolahinia, Milad Bastami, Mohammadali M. Shoja, Sepideh Zununi Vahed, Mohammadreza Ardalan

**Affiliations:** ^1^Kidney Research Center, Tabriz University of Medical Sciences, Tabriz, Iran; ^2^Research Center for Pharmaceutical Nanotechnology, Biomedicine Institute, Tabriz University of Medical Sciences, Tabriz, Iran; ^3^Noncommunicable Diseases Research Center, Fasa University of Medical Sciences, Fasa, Iran; ^4^Clinical Academy of Teaching and Learning, Ross University School of Medicine, Miramar, FL, USA

## Abstract

Idiopathic nephrotic syndrome (INS) is an important primary glomerular disease characterized by severe proteinuria. Evidence supports a role for T cell dysfunction in the pathogenesis of INS. Glucocorticoids are the primary therapy for INS; however, steroid-resistant NS (SRNS) patients are at a higher risk of drug-induced side effects and harbor poor prognosis. Although the exact mechanism of the resistance is unknown, the imbalances of T helper subtype 1 (Th1), Th2, and regulatory T cells (Tregs) and their cytokines may be involved in the pathogenesis of glucocorticoid responsiveness. Up to now, no confirmed biomarkers have been able to predict SRNS; however, a panel of cytokines may predict responsiveness and identify SRNS patients. Thus, the introduction of distinctive cytokines as novel biomarkers of SRNS enables both preventions of drug-related toxicity and earlier switch to more effective therapies. This review highlights the impacts of T cell population imbalances and their downstream cytokines on response to glucocorticoid responsiveness state in INS.

## 1. Introduction

Idiopathic nephrotic syndrome (INS) is a clinical definition, described by extreme proteinuria due to podocyte injury and foot process effacement. Focal segmental glomerulosclerosis (FSGS) and minimal change disease (MCD) are the two most important light microscopic pictures of this glomerular disease and the most common causes of INS in both adults and pediatrics. Despite the current lack of knowledge in a comprehensive understanding of the disease mechanism, the response to glucocorticoids and/or other immunosuppressant agents indicates the primary involvement of the immune system. The current observations are in favor of the association of T regulatory (Treg), T helper subtype 1 (Th1), and T helper subtype 2 (Th2) imbalances and their related cytokines in the pathogenesis of INS [1, 2].

The activation of the inflammation cascades is heterogeneous and diverse in FSGS or MCD. A sequential production of proinflammatory cytokines leads to a systemic inflammatory response initiated with the synthesis of IL-1 and TNF-*α* (TNF), which, in turn, escalates the generation of IL-6. The production of cytokines stimulates the formation of acute-phase proteins such as haptoglobin, haemopexin, or C-reactive protein (CRP), suPAR (soluble urokinase-type plasminogen activator receptor), *α*-1 antitrypsin, and fibrinogen in the liver. Alpha-1 antitrypsin and fibrinogen are more sensitive to IL-6 stimulation while others are generally synthesized in response to IL-1 [3]. Different studies have reported the plausible connection between cytokine production and proteinuria in INS [4, 5]. However, conflicting results have been obtained when the serum levels of the major cytokines and acute-phase proteins are measured in patients with NS [6–8].

Glucocorticoids are the standard initial pharmacological regimen in INS, which block the production of cytokines in both immune and nonimmune cells effectively and result in remissions in approximately 85-90% of pediatric cases. However, individuals exhibit different degrees of glucocorticoid responsiveness and variable patterns of relapses [9]. Glucocorticoids represent a key index of outcomes, and drug-resistant patients pose a challenge to clinicians. Furthermore, glucocorticoid dependency is observed in about 40–50% of the responders who are at high risk of therapy-associated unwanted effects [10]. Indeed, no clinical test is available to predict steroid resistance and/or dependence.

The response to glucocorticoids has been considered as the key variable in long-term outcomes of FSGS and MCD patients [11]. The potential effects of glucocorticoids highlight the possible role of cytokines in determining the drug response. SRNS patients without podocyte genetic defects may also respond to other immunosuppressive agents, such as cyclosporine, tacrolimus, and mycophenolate.

## 2. Factors Involved in SRNS

The impacts of epigenetic, pharmacogenetic, and genetic factors on the pathogenesis of SRNS have been comprehensively reviewed previously [12–15]. In the presence of podocyte cytoskeletal-related mutations, glucocorticoids are ineffective at restoring normal podocyte function. About 30% of SRNS patients have mutations in one of the podocyte-expressed genes. Circulating factors, such as serum urokinase-type plasminogen activator receptor or cardiotrophin-like cytokine 1, are another proposed pathogenic mechanism [16]. Cytokines are reported to modulate the glucocorticoid responses in NS [17–19]. In the following sections, we provide reported articles linking imbalanced T cell populations and their dysregulated cytokines to SRNS.

## 3. Cytokines Affect the Responses to Glucocorticoid Therapy

Because of the controversial reports regarding the cytokine patterns of Th1/Th2, subtypes, and glucocorticoid response, studies are aimed at introducing these possible biomarkers [20, 21]. In the following sections, we focus on the impacts of T cell population imbalance and its downstream cytokines on SRNS.

### 3.1. T Cell Population Imbalance in SRNS

Despite conflicting evidence, the imbalance between Th1, Th2, and Treg cells has been associated with the incidence of SRNS. If glucocorticoids mediate alterations in T cells' population and their cytokine profile, then steroid-sensitive NS (SSNS) and SRNS patients should have differences in their T cell populations. It has been demonstrated that Th1/Treg and Th2/Treg ratios are higher in SRNS compared to SSNS patients and healthy individuals, while Th1/Th2 ratios are similar among the groups. A higher ratio of Treg in comparison with Th1 and Th2 is connected with glucocorticoid sensitivity, while the reverse ratio is associated with SRNS [22]. Guimarães et al. made a study on a group of children with INS (steroid-sensitive (16 boys/9 girls) and steroid-resistant 8/6) and 10 healthy controls. They observed downregulated levels of adhesion molecules (integrin, CD18) and higher levels (48%) of Treg (TCD4^+^CTLA-4^+^ FoxP3^+^) in the steroid-sensitive group [23]. NS patients who are more prone to relapse or do not respond to glucocorticoids show an immunological switching from Th2 to Th1 [24]. In line with these findings, serum cytokines shift toward the Th1 pattern in FSGS patients [24]. Additionally, in a study on a group of INS children (29 SSNS and 14 SRNS children, aged between 2 and 19 years), higher levels of Th1 cytokines (e.g., IL-2) have been found in their serum and urine samples, whereas elevated Th2-related cytokine (i.e., IL-4) generation was associated with long-term remission [5]. However, both glucocorticoid sensitive and resistant patients show similar levels of Th1- and Th2-associated cytokines; these differences might be due to different lymphocyte stimuli [4]. Stachowski and coworkers also reported similar results and concluded that the CD4^+^ T cell-related cytokine pattern and the distribution of particular T cell subsets, including suppressor-effector (CD45RA^+^CD8^+^), suppressor-inducer (CD45RA^+^CD4^+^), and memory cells (CD45RO^+^CD4^+^), might predict the patients' sensitivity to glucocorticoids at the onset of NS [25]. The importance of the Th1/Th2 balance has been confirmed by increased levels of Th1 cytokines (including IL-2, soluble IL-2 receptor (sIL-2R), and IFN-*γ*) in SSNS patients during relapse [26]. Hence, assessing the balance of Th1/Th2 could be valuable in predicting glucocorticoid responsiveness.

Effective glucocorticoid therapy has been shown to restore the functional balance of the Th-17/Treg population in MCD patients [27]. Moreover, primary glucocorticoid therapy has reduced CD8^+^T, Th2, and CD4^+^ Th1 cells in NS patients. Accordingly, glucocorticoid therapy effectively diminishes CD8^+^T, Th2, and CD4^+^ Th1 cells in new-onset pediatric NS cases [28].

Response to glucocorticoid therapy in children with NS is influenced by the levels of IL-13 and TNF-*β* (lymphotoxin-alpha). Elevated levels of TNF-*β* are observed in SRNS patients after treatment while SSNS cases developed higher levels of IL-13. Increased levels of IL-13 may be in connection with TNF-*β* downregulation in SSNS patients since the latter is suppressed via Th2 cytokines [29]. Interaction between TNF receptor and soluble lymphotoxin-alpha promotes inflammatory responses. T cell deviation towards the Th2 population in NS patients might also be linked to the overproduction of IL-13. These findings propose that Th1-dominant patients might develop glucocorticoid-resistance, while increased IL-13 and Th2 phenotypes are in favor of a satisfactory outcome, and glucocorticoid responsiveness. Therefore, alteration in Th1 and Th2 populations and subsequent changes in IL-13/TNF-*β* cytokines balance substantially affect NS pathophysiology in children [29].

### 3.2. T Cell Resistance to Glucocorticoids

Particular mediators influence T cell resistance to glucocorticoids. For example, IL-2 and IL-4 promote lymphocyte glucocorticoid resistance during an in vitro study [30]. In addition, nuclear factor-*κ*B (NF-*κ*B) and transcription factor activator protein-1 (AP-1) are pivotal mediators of proinflammatory cytokine generation and have been found to interfere with glucocorticoid functions on T cells [31]. In this context, the glucocorticoid receptor *α* (GR*α*) suppresses AP-1 activity via direct protein-protein interaction with a c-Jun subunit of the AP-1 family [32]. Interestingly, it is documented that AP-1 modulates the structure of basal chromatin and increases the accessibility of GR and its binding to proinflammatory genes [33]. Hence, it appears that the interactions between AP-1 and glucocorticoids are far more complicated.

#### 3.2.1. NF-*κ*B Signaling

NF-*κ*B is a transcription factor that regulates the transcription of genes participating in inflammation. Sun and colleagues reported that the overexpression of NF-*κ*B in the juvenile Sprague-Dawley rat model of nephrotic syndrome induces the expression of inflammatory cytokines (IL-1 and IL-6), increases blood urea nitrogen and creatinine levels, and exacerbates renal injury [34]. NF-*κ*B as a member of the Rel family contains two subunits (p50 and p65) [35]. The binding of NF-*κ*B to the endogenous I*κ*B family proteins makes it inactive. The release of NF-*κ*B from I*κ*B occurs upon antigenic stimulation and subsequent phosphorylation of I*κ*B via I*κ*B kinases *α* and *β*. SRNS patients have a lower level of NF-*κ*B p65 subunit in the whole-cell lysates, prepared from the peripheral mononuclear blood cells (PMBC) compared to glucocorticoid-sensitive cases [36]. Both lower levels of NF-*κ*B p65 and GR*α* are connected with poor glucocorticoid responses in some patients with INS. This difference is more prominent in those experiencing relapses [36]. However, both SSNS and SRNS patients express similar levels of the p50 subunit. The translocation of the NF-*κ*B p50 subunit into the nucleus is essential for the interaction of NF-*κ*B with glucocorticoids, and the absence of such translocation impairs the ability of GRs to inhibit immune functions and NF-*κ*B transcriptional activity, inducing glucocorticoid resistance [31, 32].

The expression of IL-2 is also increased during the relapse of both SSNS and SRNS patients in comparison with controls. These results indicated alterations in the T cell populations between untreated SRNS and SSNS patients. The upregulation of IL-2 and down-regulation of NF-*κ*B p65 subunits are possible mechanisms of glucocorticoid resistance in NS [37]. It has been reported that three mechanisms are involved in this process. First, the absence of required protein-protein interactions, especially among GR*α* and p65 subunits. Second, disturbances in nuclear export of NF-*κ*B dimers, and third plunged affinity of NF-*κ*B for the glucocorticoid-stimulated leucine zipper that acts as an inhibitor of NF-*κ*B nuclear translocation [37]. In SRNS patients, steroid-based treatment might fail by enhancing NF-*κ*B function, which would worsen disease by elevating transcription of inflammatory cytokines [38].

## 4. Cytokines in SRNS

**Figure 1:**
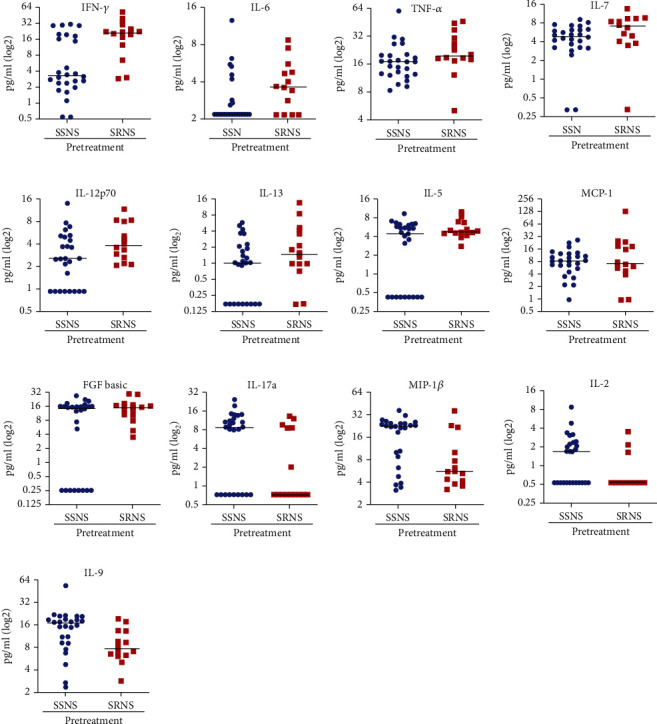
Cytokines can identify the SRNS cases before therapy. Different levels of 13 plasma cytokines were observed between SSNS versus SRNS before therapy, three of which (MCP-1, IL-9, and L-7) had values to discriminate SRNS from SSNS prior to glucocorticoid therapy with ROC value = 0.84, 0.64 sensitivity, and 0.84 specificity. FGF: fibroblast growth factor; MCP-1: monocyte chemoattractant protein-1; MIP-1*β*: macrophage inflammatory protein-1*β*; SSNS: steroid-sensitive nephrotic syndrome; SRNS: steroid-resistant nephrotic syndrome; TNF: tumor necrosis factor; ROC: receiver operating characteristic. Adapted from Ref. [28] with permission. The reference [28] article is available under the Creative Commons CC-BY-NC-ND license.

The prevalence of relapses in NS has been associated with the serum levels of particular cytokines (Table 1). Some researchers have attempted to identify urinary, plasma, and salivary cytokine-based biomarkers for SRNS in children [39–41]. Both SSNS and SRNS patients have shown suppressed levels of IL-5, IL-7, IL-13, IFN-*γ*, and TNF after glucocorticoid administration. Furthermore, SRNS patients have been shown to have higher levels of MIP-1*β*, IL-17A, IL-5, and INF-*γ* in comparison with SSNS cases in pre- and posttreatment specimens. Agrawal et al. studied the plasma profile of cytokines in children [SSNS (*n* = 26) and SRNS (*n* = 14)] aged between 18 months and 18 years before and after (7 weeks) treatment with glucocorticoids. Using a bead-based fluorescence assay, the profiling of 27 cytokines was evaluated on a Luminex Technology platform (Waltham, MA). Different levels of 13 plasma cytokines were observed between SSNS versus SRNS before therapy. Three cytokines (IL-7, IL-9, and MCP-1) exhibited ROC (receiver operating characteristic) values of 0.846, 0.64 sensitivity, and 0.84 specificity and could differentiate children with SRNS from those with SSNS at the disease onset. Furthermore, their results detected significant reductions in cytokine levels (e.g., IFN-*γ*, TNF, IL-5, IL-7, and IL-13) in response to glucocorticoid treatment in SSNS compared to SRNS patients. The authors proposed that glucocorticoid therapy decreases cytokine production by CD4^+^ Th1 cells, Th2 cells, and CD8^+^ cells in children with new-onset NS [28] (Figure 1).

Increased IL-8 concentration has been associated with relapses in NS [42] and antibodies against IL-8 could neutralize the ability of mononuclear cells to trigger albuminuria in the Wistar rat model [43]. Moreover, surged amount of IL-1*β*, IL-6, and IL-8 has been observed in INS relapses compared to healthy controls or remission in children [44]. In addition, IL-4, IL-6, and TNF polymorphisms have been in connection with glucocorticoid responsiveness in INS children [45]. The activation of TGF-*β*1 has been reported in SRNS cases, which further develop chronic kidney disease (CKD). FSGS patients have shown higher levels of urinary TGF-*β*1 compared to MCD patients. However, urinary TGF-*β*1 has not been validated as a glucocorticoid responsiveness biomarker [19]. Elevated serum levels of IL-6, haptoglobin, and haemopexin are also independent markers of glucocorticoid resistance in FSGS and MCD patients [3].

T cell expressing inflammatory cytokines, plasma macrophage migration inhibitory factor (MIF), and urinary MCP-1 are increased during persistent proteinuria in pediatric SRNS [41]. The role of glomerular macrophages and the underlying mechanism of macrophage-related glucocorticoid resistance have not been clarified. The substantial connection between urinary MCP-1 and IL-6 or interferon-inducible protein-10 (IP-10) suggests that the MCP-1-stimulated macrophages can generate IL-6 or IP-10 after recruitment to the glomeruli, which might then lead to tissue damage and enrollment of other immune cells [46, 47].

### 4.1. MIF

MIF has been considered as a suitable marker for glucocorticoid responsiveness among 48 evaluated cytokines. According to cytokine analysis, the increased plasma concentrations of MIF (cutoff concentration of MIF > 501 pg/ml) at diagnosis could identify NS children at high risk of glucocorticoid resistance. Low levels (MIF mean concentration 124.5 pg/ml in healthy controls vs. 466.1 pg/ml in INS patients) of this cytokine could also successfully discriminate INS patients from controls [48]. MIF displays proinflammatory activities as a result of interactions with T cells and macrophages. Glucocorticoids decrease the formation of inflammatory mediators; however, they accelerate MIF release from T cells and macrophages [49]. Then, MIF counterregulates the suppressor effects of glucocorticoids on proinflammatory cytokines [50]. Although the underlying mechanisms are not completely known, it has been postulated that MIF interferes with the function of glucocorticoid under an inflammatory condition mediated by NF-*κ*B-dependent manner. Glucocorticoids prevent the NF-*κ*B activation through the induction of I*κ*B*α* synthesis, whereas MIF enhances the translocation of NF-*κ*B to the nucleus [51]. Furthermore, MIF potently induces the extracellular signal-regulated kinase- (ERK-) 1 and ERK-2 pathways, which in turn, activate the intracellular isoform of phospholipase A2 (PLA2) and lead to the liberation of arachidonic acid [52]. Glucocorticoids are recognized blockers of PLA2 stimulation, and this impact is countered by MIF. Additionally, to suppress the transcription of proinflammatory genes, glucocorticoids can raise the degradation of these mRNAs; moreover, this has been revealed to be linked to the inhibitory effect of MIF on glucocorticoids. Although there are inadequate data to elucidate the proinflammatory functions of MIF entirely, the mentioned mechanisms could describe its impact on glucocorticoid-related immunosuppression [48, 53].

### 4.2. TNF

In kidney glomeruli of patients with FSGS/SRNS, activation of the TNF pathway was observed [54]. TNF is an inflammatory cytokine produced by infiltrating/circulating macrophages and monocytes. The proposed TNF mechanisms of action includes (1) leukocyte recruitment to the glomerular damage site, (2) stimulation of growth factors and cytokines, and (3) generation of oxygen radicals. Consequently, glomerular endothelial damage, apoptosis, and albumin permeability could be the result of those TNF-mediated adverse effects [55]. The intrinsic activation of the TNF signaling pathway leads to podocyte damage that can be reversed by the TNF blockader [54].

### 4.3. Suppressors of Cytokine Signaling

Suppressors of Cytokine Signaling (SOCS) prohibit Signal Transducer and Activator of Transcription (STAT) phosphorylation via blocking Janus Kinases (JaKs), and the effects of glucocorticoids on the JaK/STAT signaling cascade in children with SRNS and SSNS have been investigated. Accordingly, IL-6, IL-20, SOCS3, and SOCS5 were significantly higher in plasma samples of SRNS patients in comparison with SSNS cases. Moreover, the authors suggested the potential role of SOCS3 and SOCS5 mRNA levels as predicting factors of glucocorticoid resistance in patients with NS [56]. Furthermore, substantial lower methylation of one region of the SOCS3 promoter was observed in SRNS participants versus SSNS and normal controls [56, 57].

### 4.4. Other Cytokines

The activation of T lymphocytes and release of IFN-*γ*, IL-4, and IL-2 have been seen in SSNS children with relapse [7]. The plasma level of IL-8 has significantly been in connection with IL-4 and IL-13 in all stages of SSNS in children. Likewise, during the active phase, increased levels of IL-13, IL-4, TNF, and IgE were significantly seen in pediatric SSNS compared to patients in remission and controls [58]. It is deemed that a type-2 cytokine production succeeds in children with active SSNS, and this kind of immune response is closely correlated with the expression of IL-18 [6]. Moreover, serum levels of IL-18 are associated with both IL-4 and IL-13 in pediatric SSNS patients [59]. However, it is also reported that increased levels of IL-18 after therapy can be involved in the SRNS development [60].

## 5. Treatment

The goal of SRNS therapy is inducing complete remission; however, even partial remission may have clinical benefits. For cases with nongenetic-based SRNS, treatment with calcineurin inhibitors (tacrolimus and ciclosporin) is the standard of care therapy and 70% of them attain a partial or complete remission. The renin-angiotensin inhibitors as antihypertensive and antiproteinuric are quintessential for decreasing proteinuria [61]. Proinflammatory cytokines derived from immune cells promote the formation of angiotensin II (Ang II) both systemically and locally. Production of angiotensinogen by inflammatory cytokines is suggested as a key mechanism for the development of Ang II-dependent high blood pressure [62]. Nonresponding patients to calcineurin inhibitors or immunosuppressives are at risk for ESRD [61].

Epigenetic modification by targeting histone deacetylases (HDACs) are a promising therapeutic approach in NS. Histone deacetylase inhibitors (HDACi) play an important role in treating CKD due to their anti-fibrotic, anti-inflammatory, and immunosuppressive activities. HDACi inhibits HDACs, remodels the structure of proteins in transcription factor complexes, and causes modifications in gene transcription by removing the acetyl groups from the lysine amino acid on histone. Thus, HDACi enhances chromatin condensation and exerts a repressor effect on transcription. It is a promising intervention for targeting glomerular sclerosis and fibrosis as important pathologic features of fibrosis and CKD progression both in FSGS and INS patients. Moreover, evidence from various research has demonstrated an irregular expression of HDACs involved in renal fibrosis and glomerulosclerosis which are common pathological features of NS [63]. A combination of HDACi, vorinostat with an ACE inhibitor benazepril in an animal model of nephropathy could significantly reduce proteinuria and kidney injury via modulating different signaling cascades such as NF-*κ*B, IL-1, TGF-*β*, MAPK, and apoptosis machinery [66].

## 6. Conclusion

Alterations in cytokine patterns in INS may contribute to proteinuria and glomerular injury and influence therapeutic interventions. Thus, the identification of distinct cytokines as novel biomarkers of SRNS at the early diagnosis can benefit patients by both enabling the prevention of glucocorticoid toxicity and directing to earlier switch to more effective therapeutic options. Understanding the molecular mechanisms involved in SRNS and the development of molecular-based diagnosis and predictive biomarkers would have a significant value in the management of SRNS patients in years to come.

## Figures and Tables

**Table 1:** Summary of articles evaluated the levels of cytokines in clinical samples of patients with SRNS.

		Participants	Age	Sample	Cytokines	Results	Ref.
1	Children	SS: 26 SR: 14	SS: 5.7 ± 0.7SR: 9.5 ± 1.0	Plasma	A panel of 27 cytokines	In children with new-onset NS, glucocorticoid therapy decreases the levels of plasma cytokines secreted by CD8^+^, CD4^+^ TH1, and TH2 cells. Moreover, MCP-1, IL-9, and L-7 could predict SRNS prior to glucocorticoid therapy at disease presentation.	[28]
2	Children	C: 20 SS: 19 SR: 7	C:11.9 ± 2.5SS: 8.8 ± 3.9SR: 9.9 ± 5.5	Saliva	IL-1*β*, IL-4, IL-6, IL-8, and IFN-*γ*	The studied cytokines were not able to discriminate SRNS from SSNS or the relapse from remission states.	[40]
3	Children	SS: 29 SD: 24 SR: 9	SS: 4.3 (2–11) SD: 3.2 (1–13) SR: 8.5 (2–17)	Plasma	48 cytokines	Plasma levels of MIF can identify cases at high risk of SRNS. A cutoff MIF concentration of more than 501 pg/ml could discriminate SRNS cases with 71.4% specificity and 85.7% sensitivity.	[48]
4	Children	Remission: 9 SS: 6 SR: 4	5.9 (3.0–14.4)	PBMCs	18551 genes	In the SSNS group, the gene expression profile was enriched in genes relating to TGF-*β*1 signaling, IL-4 and IL-6, targets of FoxP3 in T lymphocytes, p53 signaling, and the cell cycle.	[21]
5	Children	C: 12 SR:12 SS: 12	C: 12.9 ± 4.3 SR: 12.3 ± 5.5 SS: 14.1 ± 4.7	Kidney and urine	TGF-1, ICAM-1	TGF-1 in urine could differentiate between MCD and FSGS; however, it was not a biomarker of steroid responsiveness.	[19]
6	Children	C: 15 NS: 42	—	Serum	IL-13, TNF-*β*	A different Th2/Th1 reaction demonstrated by an imbalance of IL-13/TNF-*β* could play a pathophysiologic role in NS. SRNS and SSNS cases had, respectively, a higher serum TNF-*β* and IL-13 level after glucocorticoid therapy than that before treatment.	[29]
7	Children	C: 10 SR: 20 SS: 34	SR: 11.3 (4–17) SS: 10.5 (4–16)	Plasma	IL-20, IL-4R, IL-6ST, JUN, MPL, MYC, SP1 and SRC, SOCS1-5	In SRNS cases, levels of IL-20, IL-6, SOCS5, and SOCS3 were elevated after 6 weeks of treatment with steroids compared to control and SSNS groups. Increased expressions of SOCS3 and SOCS5 mRNAs may predict early resistance to steroids.	[56]
8	Children	C: 5 SR: 8 SS: 8	—	CD4^+^ T cell	IFN-c, NF-*κ*B, AP-1	There were significant increase in IL-2 expression and decrease in the p65 subunit of NF-*κ*B in T cells from SRNS patients.	[37]
9	Adults	SRNS/FSGS: 96	22 ± 13	Serum, immortalized thermosensitive human podocytes (clone AB8/13)	TNF-*α* pathway genes	In podocytes of FSGS patients, activation of TNF-*α* pathway genes happens.	[54]
10	Adults	SS: 50 SR: 27	C: 50 SS: 39.8 ± 19.2 SR: 45.3 ± 18.2		IL-6, IL-1, TNF, IFN-*γ*, CRP, suPAR, haemopexin, haptoglobin	Increased levels of IL-6, haemopexin, and haptoglobin are only associated with steroid resistance in a certain group of patients. In other cases, steroid resistance is clearly unrelated to an activated inflammatory response. Multivariate analyses indicated that the levels of these 3 inflammatory factors are independent predictors of SR.	[3]

C: control; SD: steroid dependent; SS: steroid sensitive, PBMCs: peripheral blood mononuclear cells; SRNS/FSGS: steroid-resistant nephrotic syndrome/Focal segmental glomerulosclerosis; MCD: minimal change disease; TNF-*α*: tumor necrosis factor-*α*; IFN-*γ*: interferon-gamma; NF-*κ*B: nuclear factor-*κ*B; AP-1: activating protein-1; IL-6: interleukin-6; suPAR: soluble urokinase-type plasminogen activator receptor; CRP: C-reactive protein.

## Data Availability

No original data were used in this study.
